# Methodological Considerations in Multilevel Comparative Analyses of Robotic Versus Video-Assisted Thoracoscopic Lung Resection

**DOI:** 10.1093/icvts/ivag158

**Published:** 2026-05-25

**Authors:** Sarbari Swaika, Archana Dhyani, Rajnish Kumar, Heena Arora

**Affiliations:** Department of Emergency Medicine, Dr. D. Y. Patil Medical College, Hospital and Research Centre, Dr. D. Y. Patil Vidyapeeth (Deemed-to-be-University), Pimpri, Pune 411018, India; Faculty of Pharmaceutical Sciences, Graphic Era Hill University, Dehradun, 248002, India; Centre for Promotion of Research, Graphic Era Deemed University, Dehradun, 248002, India; Department of Pharmaceutics, Noida Institute of Engineering & Technology (Pharmacy Institute), Greater Noida, 201306, India; Centre of Research Impact and Outcome, Chitkara University, Rajpura 140417, Punjab, India

**To The Editor,** 

Nemoto et al.^1^ reported a large national comparison of robotic-assisted and video-assisted thoracoscopic lobectomy or segmentectomy using the Japanese DPC database and should be commended for using multilevel models, reporting both relative and absolute effect measures, and conducting several sensitivity analyses in a clinically important setting. Their findings are therefore informative, but several methodological features merit caution when interpreting the reported association between robotic surgery and postoperative ventilation.

First, the main positive signal rests on postoperative mechanical ventilation, an outcome identified from administrative codes rather than detailed clinical adjudication. In such settings, coding may capture heterogeneous scenarios, including transient ventilatory support, planned postoperative management, or true respiratory failure, and the manuscript itself suggests substantial between-hospital variation in ventilation practice.^1^ When outcome definitions are sensitive to institutional protocols, residual centre-level differences may remain even after random-intercept modelling.

Second, although modified Poisson regression with robust variance is a reasonable approach for common binary outcomes, causal interpretation still depends on adequate control of confounding.^2^ Here, the robotic group appeared systematically healthier at baseline, with less smoking exposure, earlier stage disease, fewer comorbidities, and a markedly different distribution of lobectomy and segmentectomy. Because the DPC database lacks granular variables such as pulmonary function, tumour size, conversion to thoracotomy, surgeon experience, and perioperative ventilator strategy, important confounding by indication is likely to persist despite multivariable adjustment and weighting.

Third, the sensitivity analysis that additionally adjusted for analgesic method and anaesthesia duration deserves careful interpretation. These variables may plausibly lie on the causal pathway between operative approach and postoperative respiratory outcomes. Adjustment for intermediates can bias estimation of the total effect rather than clarify it, and similarity of estimates before and after such adjustment should not be taken as evidence that indirect effects were negligible.^3^

Finally, the primary analysis relied on complete-case exclusion of a substantial proportion of otherwise eligible cases, while the justification for the missing-data mechanism remained limited. Multiple imputation was appropriately explored as a sensitivity analysis, but complete-case estimates may still be vulnerable if missingness was related to patient complexity or institutional documentation patterns.^4^

Taken together, this study contributes useful real-world data, but its results may be better viewed as evidence of an association shaped by case selection, institutional practice, and residual confounding, rather than definitive comparative safety. Greater attention to end-point specificity, missing-data assumptions, and the causal role of post-exposure variables would strengthen inference in future analyses.

## Data Availability

Not applicable.
